# Expanding the Benefits of *Tnt1* for the Identification of Dominant Mutations in Polyploid Crops: A Single Allelic Mutation in the MsNAC39 Gene Produces Multifoliated Alfalfa

**DOI:** 10.3389/fpls.2021.805032

**Published:** 2021-12-24

**Authors:** Cintia Jozefkowicz, Cristina Gómez, Ariel Odorizzi, Anelia Iantcheva, Pascal Ratet, Nicolás Ayub, Gabriela Soto

**Affiliations:** ^1^Instituto de Agrobiotecnología y Biología Molecular (INTA-CONICET), Buenos Aires, Argentina; ^2^Instituto de Genética (IGEAF), Instituto Nacional de Tecnología Agropecuaria (INTA), Buenos Aires, Argentina; ^3^Estación Experimental Agropecuaria Manfredi, Instituto Nacional de Tecnología Agropecuaria (INTA), Córdoba, Argentina; ^4^AgroBioInstitute, Agricultural Academy, Sofia, Bulgaria; ^5^Université Paris-Saclay, INRAE, CNRS, Université d’Évry, Institute of Plant Sciences Paris-Saclay (IPS2), Orsay, France; ^6^Université de Paris, CNRS, INRAE, Institute of Plant Sciences Paris Saclay (IPS2), Orsay, France

**Keywords:** transformation, *Tnt1*, dominant mutation, polyploidy, alfalfa

## Abstract

Most major crops are polyploid species and the production of genetically engineered cultivars normally requires the introgression of transgenic or gene-edited traits into elite germplasm. Thus, a main goal of plant research is the search of systems to identify dominant mutations. In this article, we show that the *Tnt1* element can be used to identify dominant mutations in allogamous tetraploid cultivated alfalfa. Specifically, we show that a single allelic mutation in the MsNAC39 gene produces multifoliate leaves (*mfl*) alfalfa plants, a pivot trait of breeding programs of this forage species. Finally, we discuss the potential application of a combination of preliminary screening of beneficial dominant mutants using *Tnt1* mutant libraries and genome editing *via* the CRISPR/Cas9 system to identify target genes and to rapidly improve both autogamous and allogamous polyploid crops.

## Introduction

Legume crops fix nitrogen symbiotically by interacting with soil nitrogen-fixing rhizobia, producing high-protein foods and reducing the use of nitrogen fertilizers derived from fossil fuel. The most important forage legume worldwide is alfalfa (*Medicago sativa*), also called the “Queen of forages” due to its high yield and quality and wide adaptation. However, traditional and modern alfalfa breeding programs are limited by the intrinsic features of the species itself.

Cultivated alfalfa is an allogamous perennial tetraploid species which displays high levels of self-incompatibility and extreme inbreeding depression (Dieterich Mabin et al., 2021). Because of this polyploid nature and particular reproductive behavior, an alfalfa cultivar commonly involves a large number of genetically and phenotypically heterozygous parental plants. Then, the production of genetically modified alfalfa cultivars needs the introgression of transgenic or gene-edited traits into elite heterogeneous populations, independently of the agronomic quality of the regenerative clone used in alfalfa transformation.

Empirically, only two introgression approaches have been able to bypass the intrinsic limitations of the polyploid and outcrossing nature of alfalfa: the dihomogenic and supertransgene process described by Forage Genetics Inc. (McCaslin et al., 2002) and that described by the research group of Dr. Gabriela Soto (Jozefkowicz et al., 2018). These rapid and low-cost introgression processes require the use of dominant traits (e.g., herbicide tolerance transgenes), excluding the use of beneficial recessive mutations such as some alfalfa mutant events generated *via* the CRISPR/Cas9 system (Gao et al., 2018).

In the last years, the increased efficiency of the CRISPR/Cas9 system in highly regenerative alfalfa germplasms has allowed full allelic knockout of an individual gene in the T0 generation (Chen et al., 2020; Wolabu et al., 2020; Bottero et al., 2021). Naturally, this optimized system can help to validate in alfalfa the strategies found in the model species *Medicago truncatula*. As an example of this transfer, null *palm1* mutants produce pentafoliate leaves rather than wild-type trifoliate leaves in both *M. truncatula* (Chen et al., 2010) and alfalfa (Chen et al., 2020). Although the high expression of this type of beneficial traits (e.g., multifoliated leaves) obtained by the full allelic knockout of an individual alfalfa gene (e.g., *palm1*) in elite germplasms is not impossible, it requires an adaptation of the current introgression processes, a fact that increases the cost and time of production of a genetically modified alfalfa cultivar (Bottero et al., 2021).

Retrotransposon *Tnt1*, an effective mutagen, has been used as a tool to study gene function in both model (e.g., *M. truncatula*, *Arabidopsis thaliana* and wild potato) and non-model (e.g., soybean, lotus, cucumber and lettuce) autogamous species (Courtial et al., 2001; d’Erfurth et al., 2003; Mazier et al., 2007; Cui et al., 2013; Duangpan et al., 2013; Iantcheva et al., 2016). In this classical application of *Tnt1* for functional genomics studies, homozygous mutant lines generated *via Tnt1* insertions show complete loss of the function of target genes (full allelic knockout). In this article, we explore the use of *Tnt1* for the identification of beneficial dominant mutations in alfalfa. Specifically, we focus on dominant alleles, which are those that express a trait even if there is only one copy.

## Empirical Confirmation of the Utility of *Tnt1* to Identify Dominant Mutations in Alfalfa

**FIGURE 1:**
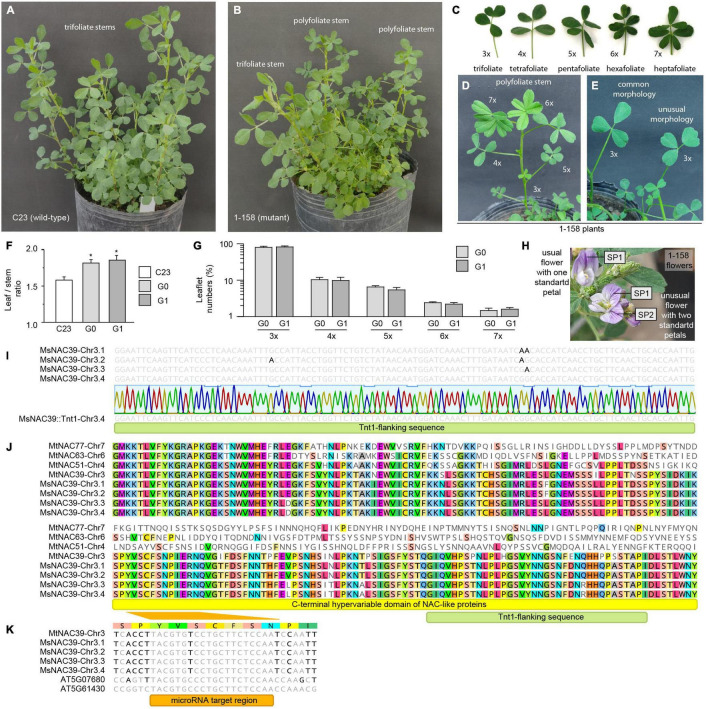
Identification of a dominant mutation conferring multifoliate leaves in cultivated alfalfa *via Tnt1*. During a screening of dominant mutations with visible phenotype from an alfalfa *Tnt1* insertion mutant library using the wild-type highly regenerative alfalfa clone C23 (García et al., 2014), we identified a mutant (1–158, *mfl*) plant displaying both usual trifoliate stems **(A,B)** and unusual polyfoliate leaves **(B)**. These mutant plants show trifoliate to heptafoliate leaves **(C)**, polyfoliate leaves with different number of leaflets **(D)**, and tetrafoliate leaves with unusual morphologies **(E)**. When comparing clonally propagated individual plants derived from the wild-type clone (C23), the original mutant clone 1–158 (G0) and its first generation offspring (G1), we observed that the *mfl* mutation induces a higher leaf-to-stem ratio **(F)** and higher numbers of leaflets **(G)**. The *mfl* mutant also shows flowers with extra petals **(H)**. In these experiments, the cuts were performed at 8 cm from the soil and plants were harvested periodically (every 60 days) to prevent their flowering and evaluate their leaf/stem ratio and number of leaflets for 2 years. The values are the average for this period. Wild-type and mutant plants were intercrossed by hand. Wild-type plants contains four different alleles of MsNAC39 and *Tnt1* is inserted into this gene on chromosome 3.4 in *mfl* mutant plants **(I)**. Multiple alignment of the partial amino acid sequences of MsNAC39 protein and its closely related proteins in *M. truncatula* (MtNAC77, MtNAC63, and MtNAC51) showing that the *Tnt1* insertion is localized in the C-terminal hypervariable domain of the MsNAC39 protein **(J)**. The microRNA target region of Arabidopsis NAC-like genes AT5G61430 and AT5G07680 is conserved in the MsNAC39 alleles **(K)**. Values are mean ± SEM (*n* = 25). Statistical analysis was carried out with Student’s *t*-test (*p < 0.05).

Due to their high protein content and high digestibility, alfalfa leaves contribute the majority of the feeding value of this forage. Thus, increasing the proportion of leaves to stems is a pivotal target of alfalfa breeding programs (Odorizzi et al., 2015). During a screening of dominant mutations with visible phenotype and Mendelian inheritance within an alfalfa *Tnt1* library performed at our lab (Supplementary Figure 1), we identified an alfalfa mutant with polyfoliated leaves. Contrary to its parental wild-type plant C23, which shows common trifoliate leaves (Figure 1A), this mutant (mutant 1–158) displays both trifoliate and polyfoliate stems (Figure 1B). These multifoliate leaves (*mlf*) plants have different numbers (3–7) of leaflets (Figure 1C) and leaf morphologies (Figures 1D–E). As expected, this *mlf* mutation produces a higher leaf-to-stem ratio (Figure 1F) and higher numbers of leaflets (Figure 1G), at least in the first generation. Moreover, around 50% of the *mlf* flowers show an increased number of petals (Figure 1H), an extremely unusual (<0.001%) trait in cultivated alfalfa. In biotechnological terms, these findings represent the first report of a dominant mutation conferring polyfoliate leaves in alfalfa and of the expanded use of *Tnt1* for the identification of this type of dominant mutations in polyploid species.

## A Mutation in the MsNAC39 Gene Produces Multifoliate Leaf Alfalfa

Because of the extreme heterozygosity of alfalfa, an individual plant (e.g., regenerative clone C23) normally possesses four different alleles of the same locus, and it is thus possible to determine the exact localization of a mutation by segregation analysis (Bottero et al., 2021). Bioinformatic analysis of the *Tnt1*-flanking sequences of the *mfl* mutant plant and its progeny showed that the *mfl* mutation colocalized with the MsNAC39 gene in chromosome 3.4 (Figure 1I). The NAC protein family is a large group of plant-specific transcriptional factors involved in the control of gene expressions related to leaf development and adaptation to environmental conditions (Liu et al., 2014; Oda-Yamamizo et al., 2016; Trupkin et al., 2019; Min et al., 2020; Challa et al., 2021). NAC transcriptional factors contain a highly conserved N-terminal DNA-binding domain and a C-terminal hypervariable domain related to the activation or repression of the transcription of specific target genes (Yamaguchi-Shinozaki and Shinozaki, 2005; Le et al., 2011; He et al., 2016; Maugarny et al., 2016). Similarly, the MsNAC39 protein and its closely related proteins (>40% amino acid identity) in *M. truncatula* (MtNAC77, MtNAC63, and MtNAC51) show a completely different C-terminal region (Figure 1J). Interestingly, the *Tnt1* element disrupts the particular C-terminal region of the NAC39 protein in *mlf* alfalfa (Figure 1J), suggesting that this mutation affects its transcriptional factor activity. In addition the Arabidopsis NAC-like proteins most related to MsNAC39 (AT5G61430 and AT5G07680; Supplementary Figure 2), together with the CUC1 and CUC2 genes (Supplementary Figure 2) are common targets of a microRNA controlling leaf and floral development (Mallory et al., 2004; Rubio-Somoza et al., 2014). An alteration in their expression leads to the development of striking floral organ phenotypes, including extra petals (Mallory et al., 2004; Baker et al., 2005) in agreement with the *mfl* alfalfa phenotype. Interestingly, the microRNA target region of AT5G61430 and AT5G07680 is highly preserved in the MsNAC39 alleles, sharing 19 of the 20 nucleotides (Figure 1K). Taken together, evolutionary and phenotypic studies suggest that MsNAC39 can play ancient and multiple roles in alfalfa ontogeny, including vegetative growth and reproductive development.

## Discussion

**FIGURE 2:**
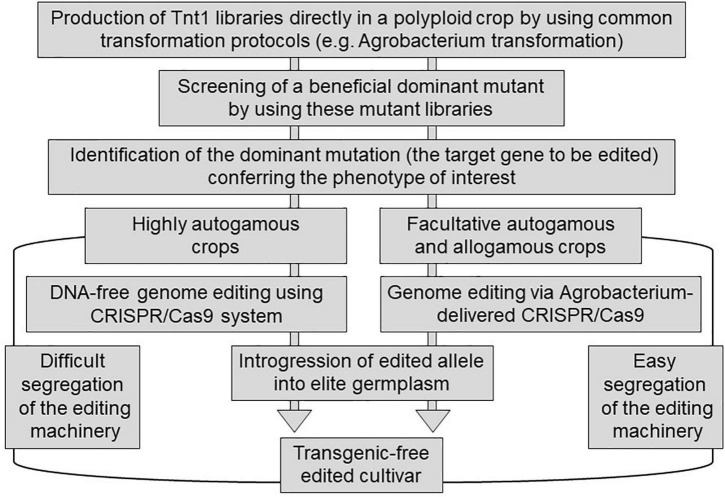
Schematic representation of the possible combination of *Tnt1* element and the CRISPR/Cas9 system to rapidly improve polyploidy crops. This picture is explained in the text.

Although there is a legal framework for the deregulation of transgenic plants, including transgenic alfalfa cultivars (Samac and Temple, 2021), the deregulation of transgenic events are long-term and expensive procedures. Specifically, *Tnt1* is a mobile element that can move around within a genome not only under regenerative conditions but also under abiotic stress conditions (Iantcheva et al., 2009), which is an undesirable trait in transgenic crops. Thus, the direct application of *Tnt1* insertions (e.g., MsNAC39:*Tnt1*) in crop production is extremely limited. In contrast, engineering plant traits using the CRISPR/Cas9 system (e.g., the induction of small indels in MsNAC39 alleles) could be an attractive strategy to improve germplasms available worldwide. However, the current knowledge of beneficial dominant mutations is very scarce, thus limiting the rational design of the generation of this type of mutation *via* the CRISPR/Cas9 system in polyploid species. Most major crop species, including soybean, wheat, maize, potato, sugarcane, coffee, cotton, alfalfa, and tall fescue, are polyploid species. In this context, *Tnt1* libraries can function as sources of beneficial dominant mutations that can be phenocopied using transgenic-free edited cultivars *via* the CRISPR/Cas9 system in these major crops with complex genetics (Figure 2). In the case of facultative autogamous and allogamous species, the editing machinery can be easily segregated during the introgression process, and then, the *Agrobacterium* transformation does not imply a limitation to produce transgenic-free commercial cultivars (Figure 2). In contrast, in the case of highly autogamous species, the use of the CRISPR/Cas9 system with economic purposes implies the search of alternative deliveries of the editing machinery to the target plant cell, such as the transfection of protoplasts with ribonucleoprotein complexes (Figure 2). The perspective of combining the *Tnt1* element and the CRISPR/Cas9 system could lead to a synergism between applied research and breeding programs, in which breeders will rapidly convert beneficial dominant mutations in improved elite germplasm varieties.

## Data Availability Statement

The original contributions presented in the study are included in the article/Supplementary Material, further inquiries can be directed to the corresponding author/s.

## Author Contributions

GS, NA, CJ, AI, and PR designed and funded the experiments. CJ and CG performed the experiments. CJ, CG, AO, AI, PR, NA, and GS helped with the experiments and data analysis. GS wrote the manuscript. All authors contributed to the article and approved the submitted version.

## Conflict of Interest

The authors declare that the research was conducted in the absence of any commercial or financial relationships that could be construed as a potential conflict of interest.

## Publisher’s Note

All claims expressed in this article are solely those of the authors and do not necessarily represent those of their affiliated organizations, or those of the publisher, the editors and the reviewers. Any product that may be evaluated in this article, or claim that may be made by its manufacturer, is not guaranteed or endorsed by the publisher.
