# Supplementary material for: Expanding the Benefits of Tnt1 for the Identification of Dominant Mutations in Polyploid Crops: A Single Allelic Mutation in the MsNAC39 Gene Produces Multifoliated Alfalfa
Source: Front Plant Sci. 2021 Dec 24;12:805032. doi: 10.3389/fpls.2021.805032 (PMC8763170; doi:10.3389/fpls.2021.805032)
Supplement: Supplementary file 1 [file Data_Sheet_1.PDF]

Figure S1

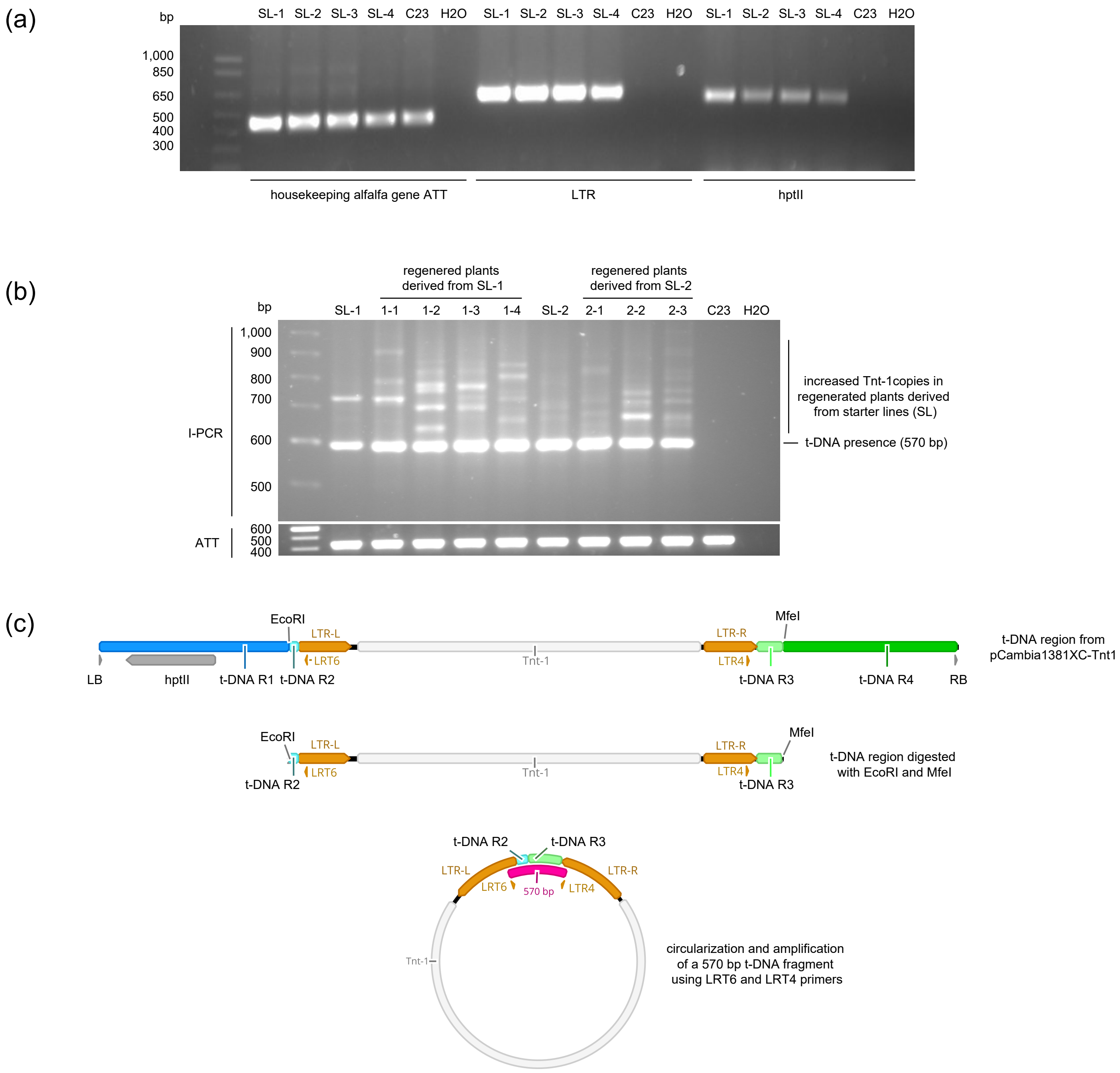

Figure S1. Production of an alfalfa Tnt-1 library. Approximately 8,500 independent insertion mutants were generated at the National Institute of Agricultural Technology (INTA), Argentina, by somatic embryogenesis using leaf explants from eleven starter lines (SL) containing from one to nine copies of Tnt-1. SL were generated by transformation of the highly regenerative clone C23 (Jozefkowicz et al., 2018;Bottero et al., 2021) with the binary vector pCambia1381XC-Tnt1 (Iantcheva et al., 2009) using hygromycin (50 mg/ml) as selective agent. The regeneration protocol described previously by INTA (García et al., 2014) was used for the induction of Tnt1 mobilization in the alfalfa genome, obtaining an increase of 5.1 new copies of Tnt1 after its passage through regeneration. Alternative protocols (e.g. using different concentrations of synthetic auxins and sucrose) displayed reduced Tnt1 mobilization and/or plant survival (mainly due to plant oxidation) in greenhouse conditions. (a) Representative analysis of the presence of t-DNA in SL by using PCR amplification of both LTR and hptII sequences. (b) Representative analysis of the presence of t-DNA in SL-1 and SL-2, and their derived regenerated plants 1-1 to 1-4 and 2-1 to 2-3, respectively, by using I-PCR amplification of a 570-bp t-DNA fragment. (c). This amplification band (570 bp) contains fragments of LTR-L, t-DNA R2, t-DNA R3 and LTR-R. PCR and I-PCR assays were performed as previously (Iantcheva et al., 2016), using the housekeeping gene AAT as control (García et al., 2014). Panel (b) also shows a representative view of the increase in the Tnt-1 copies in regenerated plants derived from SL.
