# Supplementary material for: Expanding the Benefits of Tnt1 for the Identification of Dominant Mutations in Polyploid Crops: A Single Allelic Mutation in the MsNAC39 Gene Produces Multifoliated Alfalfa
Source: Front Plant Sci. 2021 Dec 24;12:805032. doi: 10.3389/fpls.2021.805032 (PMC8763170; doi:10.3389/fpls.2021.805032)
Supplement: Supplementary file 2 [file Data_Sheet_2.PDF]

Figure S2

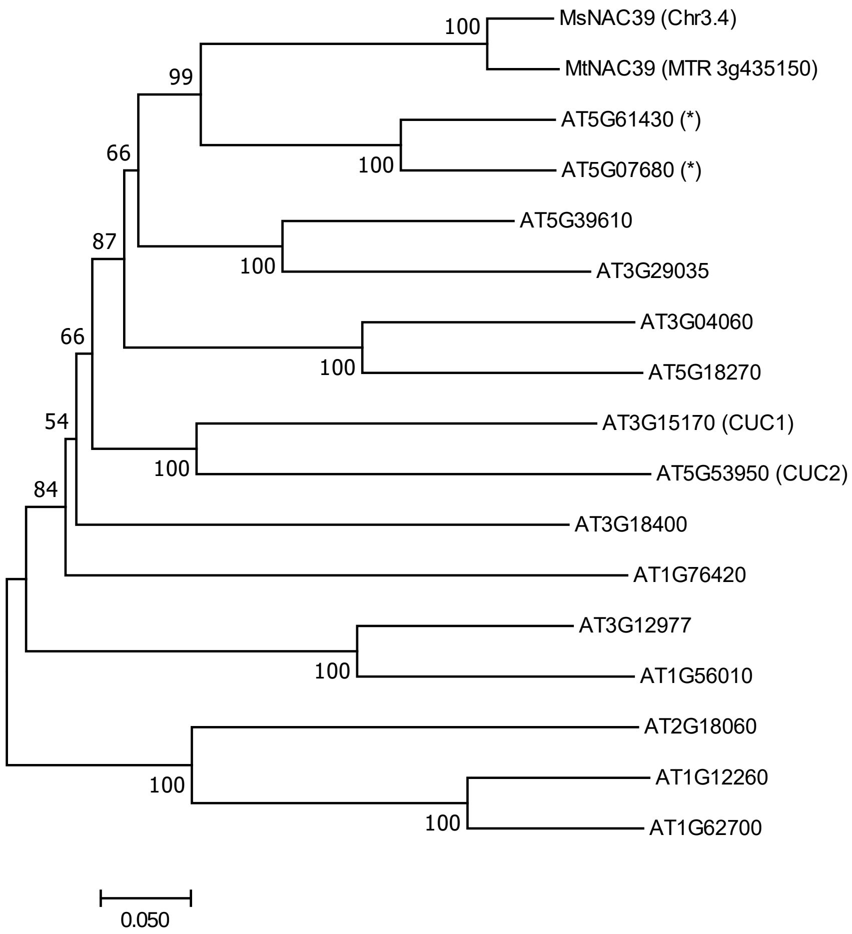


**Figure S2. Phylogenetic relationships between MsNAC39 and NAC-like proteins from *Arabidopsis thaliana.*** Protein sequences were aligned using the ClustalW program. A phylogenetic tree was constructed using the Neighbor Joining (NJ) method with genetic distances computed using the p-distance model and bootstrap analysis of 500 resamples and root on midpoint. The phylogenetic analysis was performed by using MEGA software (https://www.megasoftware.net/). This evolutionary analysis was restricted to Arabidopsis proteins with high protein identity (>30%) related to MsNAC39. Bootstrap percentages of 40% or more are indicated at the branch points. Arabidopsis proteins with higher evolutionary affinity to MsNAC39 are highlighted with asterisks.
